# Autopsy-Based Insights Into Maternal Mortality: A Retrospective Study From a Tertiary Care Center in North India

**DOI:** 10.7759/cureus.108077

**Published:** 2026-04-30

**Authors:** Arushi Verma, R Sivasankary, Shakti Bansal, Neha Soibam, Anand Kumar, Nikita Sihag, Raviprakash Meshram

**Affiliations:** 1 Forensic Medicine and Toxicology, All India Institute of Medical Sciences, Rishikesh, Rishikesh, IND

**Keywords:** autopsy, maternal death, maternal mortality, obstetric hemorrhage, postpartum deaths

## Abstract

Introduction: Maternal mortality remains a major public health concern, particularly in low- and lower-middle-income countries such as India. This retrospective, autopsy-based study was conducted to analyze the epidemiological characteristics, obstetric profile, and causes of maternal deaths at a tertiary care center in North India.

Methodology: A total of 29 confirmed maternal death cases brought for medico-legal autopsy at the Advanced Autopsy Centre, All India Institute of Medical Sciences (AIIMS), Rishikesh, between October 2018 and January 2026 were included. Data were obtained from autopsy reports, clinical records, and inquest papers and analyzed using descriptive statistics.

Results: The majority of maternal deaths occurred in women aged 20-30 years (n = 20, 69.0%), with a median age of 29 years. Most cases were from rural areas (n = 24, 82.8%) and belonged to lower middle (n = 14, 48.3%) and middle socioeconomic classes (n = 12, 41.4%). A considerable proportion of women had low educational status, with most attaining only middle or primary school education. The postpartum period accounted for the majority of deaths (n = 22, 75.9%), with all such deaths occurring within 10 days of delivery. Most women were referred from peripheral healthcare facilities (n = 24, 82.8%), and 41.4% (n = 12) were brought dead to the hospital. Direct obstetric causes accounted for 65.5% (n = 19) of maternal deaths, while 34.5% (n = 10) were due to indirect causes. Obstetric hemorrhage was the leading cause (n = 8, 27.6%), followed by sepsis (due to pregnancy-related infection) (n = 7, 24.1%) and pregnancy-induced hypertension (n = 4, 13.8%). Indirect causes included pulmonary tuberculosis (n = 2, 6.9%), pneumonia (n = 2, 6.9%), cerebrovascular thrombosis (n = 2, 6.9%), and cardiac conditions (n = 2, 6.9%).

Conclusion: The findings highlight critical gaps in timely referral, emergency transport, and postpartum care, along with the influence of socioeconomic and educational factors on maternal outcomes. Autopsy-based evaluations provide valuable insights into the accurate causes and determinants of maternal mortality. Strengthening healthcare infrastructure, improving referral systems, and addressing social determinants are essential to reducing preventable maternal deaths and accelerating progress toward achieving the Sustainable Development Goals (SDGs).

## Introduction

Maternal death is defined as the death of a woman during pregnancy or within 42 days of pregnancy termination, regardless of the pregnancy's duration and site, from any cause related to or aggravated by the pregnancy or its management, but not from accidental or incidental causes [1]. Maternal mortality is an essential indicator of a woman’s overall reproductive health in a country.

According to the latest WHO data, approximately 260,000 maternal deaths occurred worldwide in 2023. Of these, about 92% were from low- and lower-middle-income countries, with Sub-Saharan Africa alone accounting for 70% (182,000 deaths) and Southern Asia for 17% (43,000 deaths) [2]. A key measure for evaluating maternal mortality is the Maternal Mortality Ratio (MMR), which is the number of maternal deaths per 100,000 live births in a specific period. The global MMR for 2023 was estimated at 197 per 100,000 live births, marking a 40% decrease from 328 in 2000. Nonetheless, progress is still insufficient to reach Target 3.1 of the Sustainable Development Goals (SDGs), which aims to reduce the global MMR to below 70 per 100,000 live births by 2030. Achieving this target would require an annual reduction rate of nearly 15%, which is 10 times faster than the current global pace [2].

In India, the WHO estimates the MMR to be 80 in 2023 [2]. National data from the Sample Registration System (SRS), a large demographic survey managed by the Registrar General of India, provide more detailed, state-by-state information. According to the most recent SRS bulletin (2020-2022), the national MMR decreased from 93 (2019-21) to 88. Significant differences exist among states, with Kerala reporting the lowest MMR at 18, while Madhya Pradesh reported the highest at 159 [3]. These disparities highlight inequalities in access to quality maternal healthcare and reflect broader socioeconomic gaps between regions.

Women in low-income countries also face a disproportionately higher lifetime risk of maternal death. This risk is the chance that a woman aged 15-49 years will die from maternal causes, assuming a uniform risk throughout her reproductive years. Based on SRS 2020-2022 data, the lifetime risk of maternal death in India is 0.18% [3].

Autopsy-based studies are crucial for accurately identifying the causes of maternal deaths. Often, clinical documentation may be incomplete or may misclassify the cause of death, especially in complex or rapidly progressing obstetric cases. Additionally, analyzing the socio-demographic profile of the deceased helps in understanding the broader factors influencing maternal mortality. Recognizing these factors is essential for improving emergency obstetric care and formulating targeted public health strategies to reduce preventable maternal deaths [1-3]. This study is a retrospective, autopsy-based analysis of 29 maternal death cases brought for medico-legal autopsy at the Advanced Autopsy Centre at All India Institute of Medical Sciences (AIIMS), Rishikesh.

## Materials and methods

Study characteristics

This is a retrospective, cross-sectional, autopsy-based descriptive study conducted to assess the epidemiological characteristics, obstetric profile, and causes of maternal deaths, with the aim of understanding the patterns and determinants of maternal mortality. The study was conducted over a period of seven years and three months, from October 2018 to January 2026. The study was carried out at the Advanced Autopsy Centre, AIIMS, Rishikesh, a tertiary care center in North India. All cases of maternal deaths brought for medico-legal autopsy at the Advanced Autopsy Centre during the study period were included in the study. The study employed a consecutive sampling method. This is a time-bound study, which identified a total of 29 confirmed maternal death cases during the study period.

Inclusion and exclusion criteria

All confirmed maternal death cases, as defined by standard criteria [1], brought for medico-legal autopsy during the study period were included in the study. Cases not fulfilling the definition of maternal death, including accidental, suicidal, or incidental deaths unrelated to pregnancy, were excluded.

Data collection

Data were collected retrospectively from postmortem reports, clinical records (where available), and inquest papers. A structured data abstraction proforma was used to record relevant variables, including epidemiological characteristics (age, residence, socioeconomic status, and education level), obstetric profile (gestational age/postpartum period, mode of delivery, place of death, and referral status), and causes of death.

All autopsies were performed according to standard medico-legal protocols. Specimens for histopathological examination were preserved where indicated. The probable cause of death was established based on the correlation of clinical and postmortem findings and histopathological examination.

Data analysis

The collected data were entered into Microsoft Excel (Microsoft Corporation, Redmond, WA, USA) and analyzed using descriptive statistics. Results were expressed as frequencies and percentages and presented using tables and graphical representations. No inferential statistical analysis was performed due to the limited sample size.

Ethical clearance

Ethical clearance for the study was obtained from the Institutional Ethics Committee of AIIMS Rishikesh (AIIMS/IEC/25/564). As the study involved retrospective analysis of autopsy reports, clinical records, and inquest papers without interaction with living subjects, the requirement for informed consent was waived. Confidentiality of all personal identifiers was strictly maintained.

## Results

A total of 4,580 medico-legal autopsies were conducted during the study period, of which 702 cases were women. Among these, 31 women were pregnant; however, only 29 cases (0.63%) were confirmed as maternal deaths and were included in the present study.

Age

**Figure 1:**
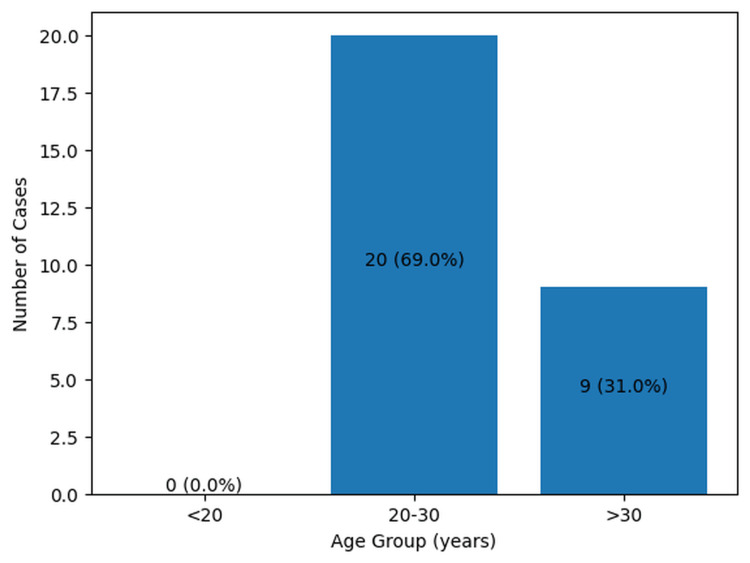
Distribution of cases by age (n=29)

The age of the deceased ranged from 20 to 43 years, with a median age of 29 years. For analytical purposes, age was categorized into three groups: <20 years, 20-30 years, and >30 years [4]. The majority of maternal deaths occurred in the 20-30 year age group (n = 20, 69.0%), followed by women aged >30 years (n = 9, 31.0%). No maternal deaths were observed among women younger than 20 years (Figure 1).

Socioeconomic status

**Figure 2:**
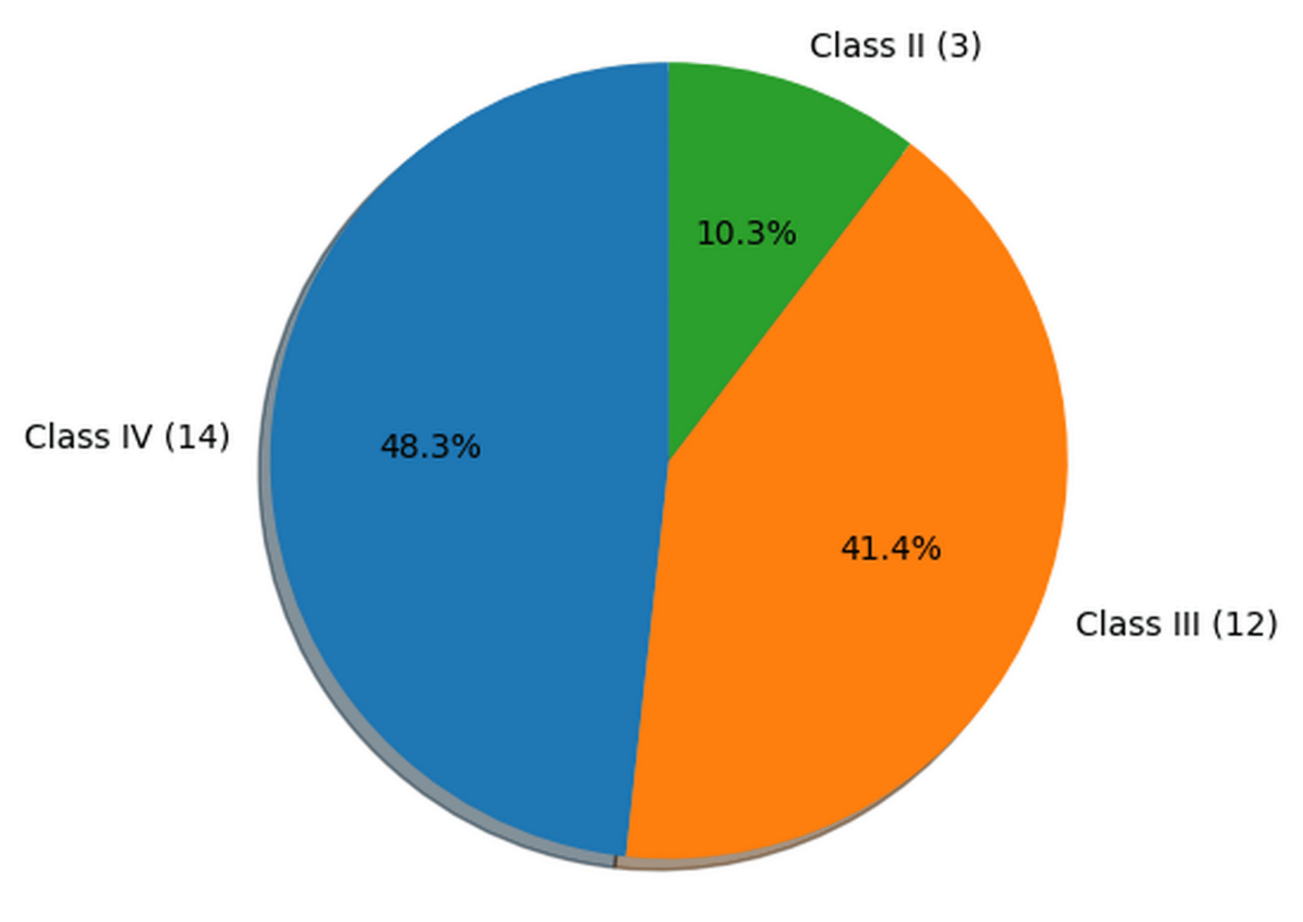
Distribution of cases by socioeconomic status using the Modified B.G. Prasad classification (latest CPI adjusted) (n=29) CPI: Consumer Price Index

Socioeconomic status was assessed using the Modified B.G. Prasad classification (latest Consumer Price Index (CPI)-adjusted), based on per capita monthly income [5]. The majority of cases belonged to Class IV (lower middle class) (n = 14, 48.3%), followed by Class III (middle class) (n = 12, 41.4%) and Class II (upper middle class) (n = 3, 10.3%). No cases were observed in the upper (Class I) or lower (Class V) socioeconomic classes (Figure 2). With respect to residence, most of the women were from rural areas (n = 24, 82.8%), while only 5 (17.2%) were from urban areas.

Education level

**Figure 3:**
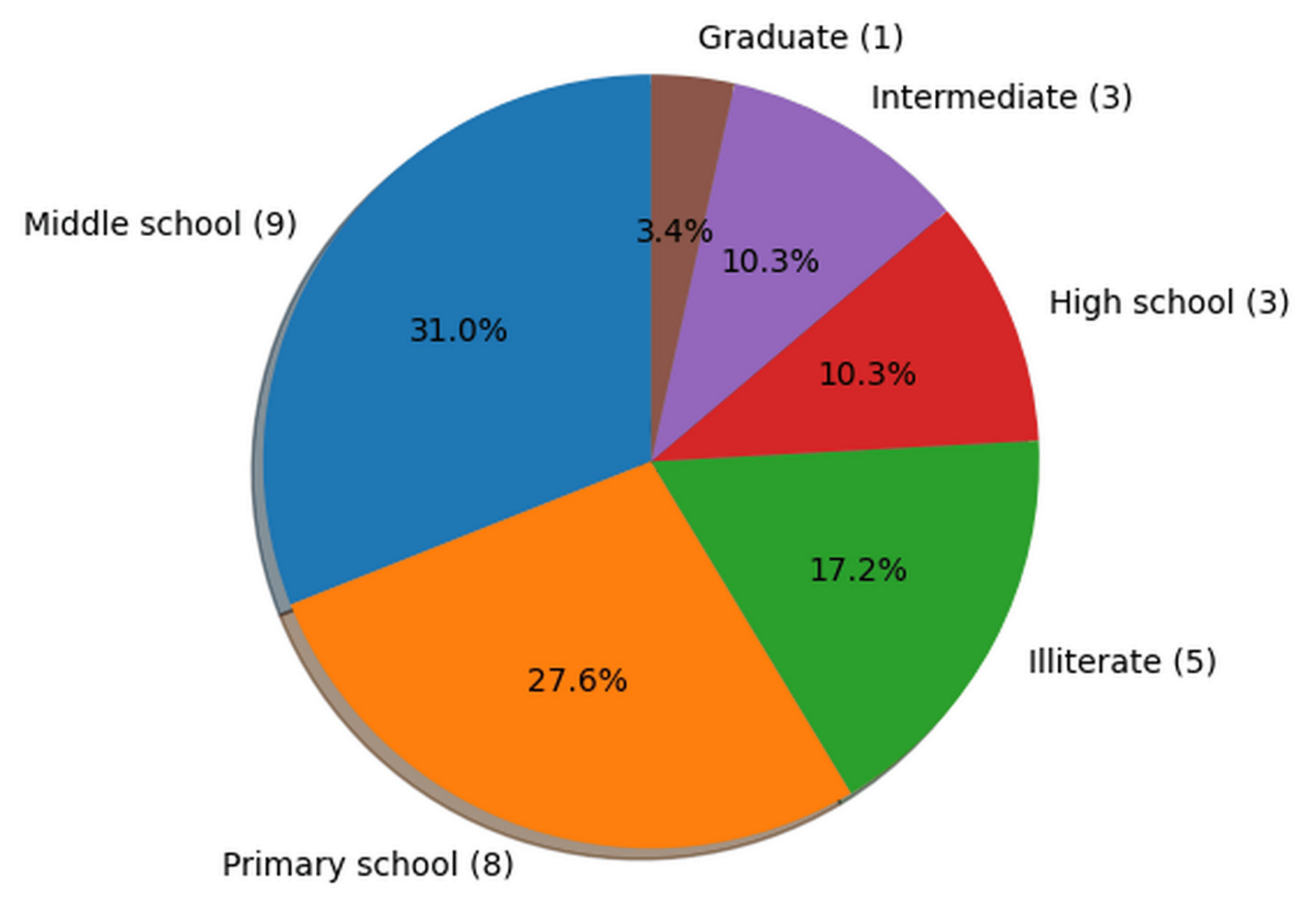
Distribution of cases by education level using the Modified Kuppuswamy scale (n=29)

Education level was classified according to the Modified Kuppuswamy scale [6]. The majority of cases had attained education up to middle school (n = 9, 31.0%), followed by primary school (n = 8, 27.6%). A considerable proportion of the cases were illiterate (n = 5, 17.2%). High school and intermediate levels of education were each observed in three cases (10.3%), while only one case (3.4%) had completed graduation (Figure 3).

Gestational age/postpartum period

**Figure 4:**
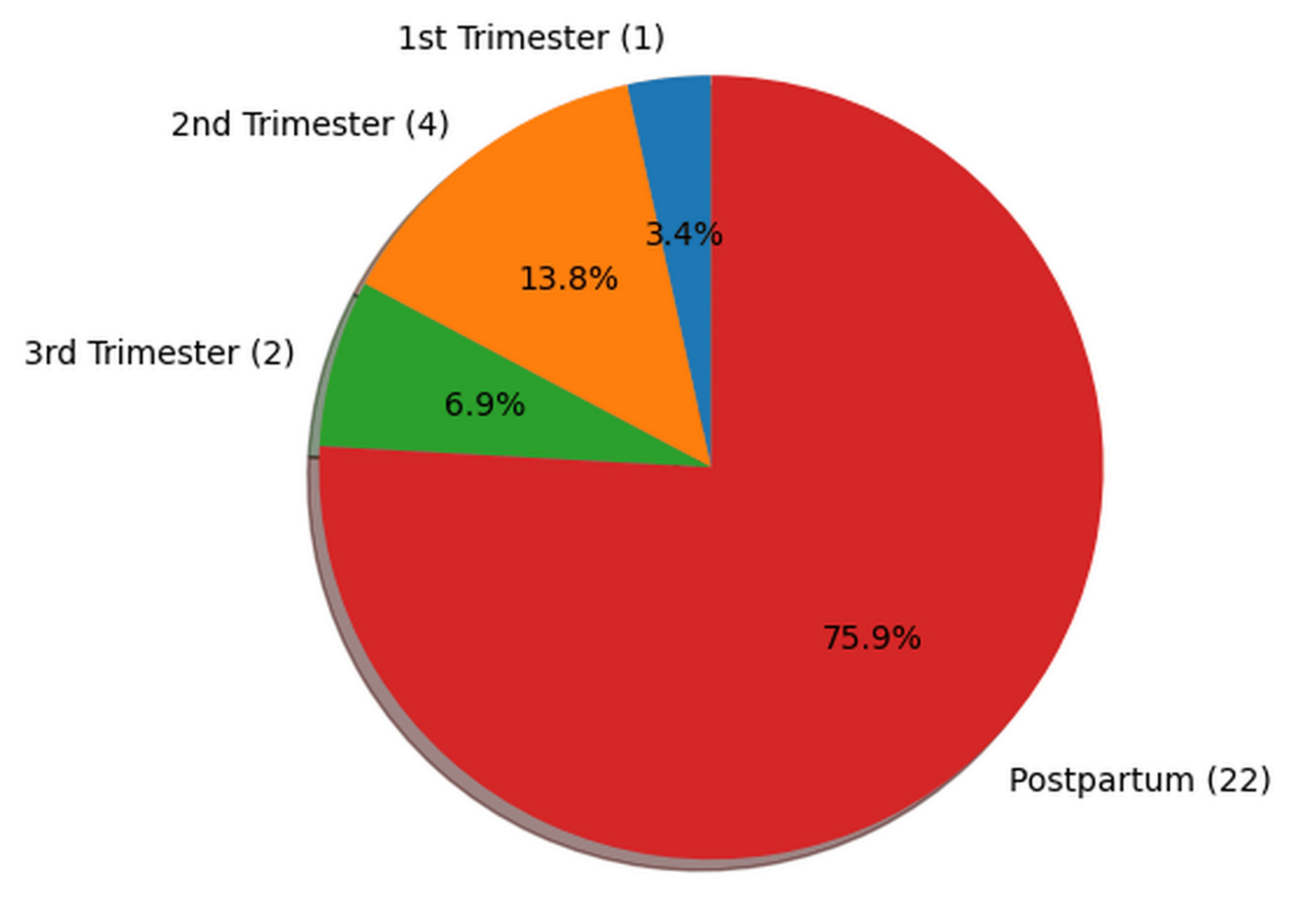
Distribution of cases by gestational age/postpartum period (n=29)

The majority of maternal deaths occurred during the postpartum period (n = 22, 75.9%), with all these cases occurring within the first 10 days following delivery. In contrast, four deaths (13.8%) occurred during the second trimester, two deaths (6.9%) during the third trimester, and one death (3.4%) during the first trimester (Figure 4).

Mode of delivery

**Figure 5:**
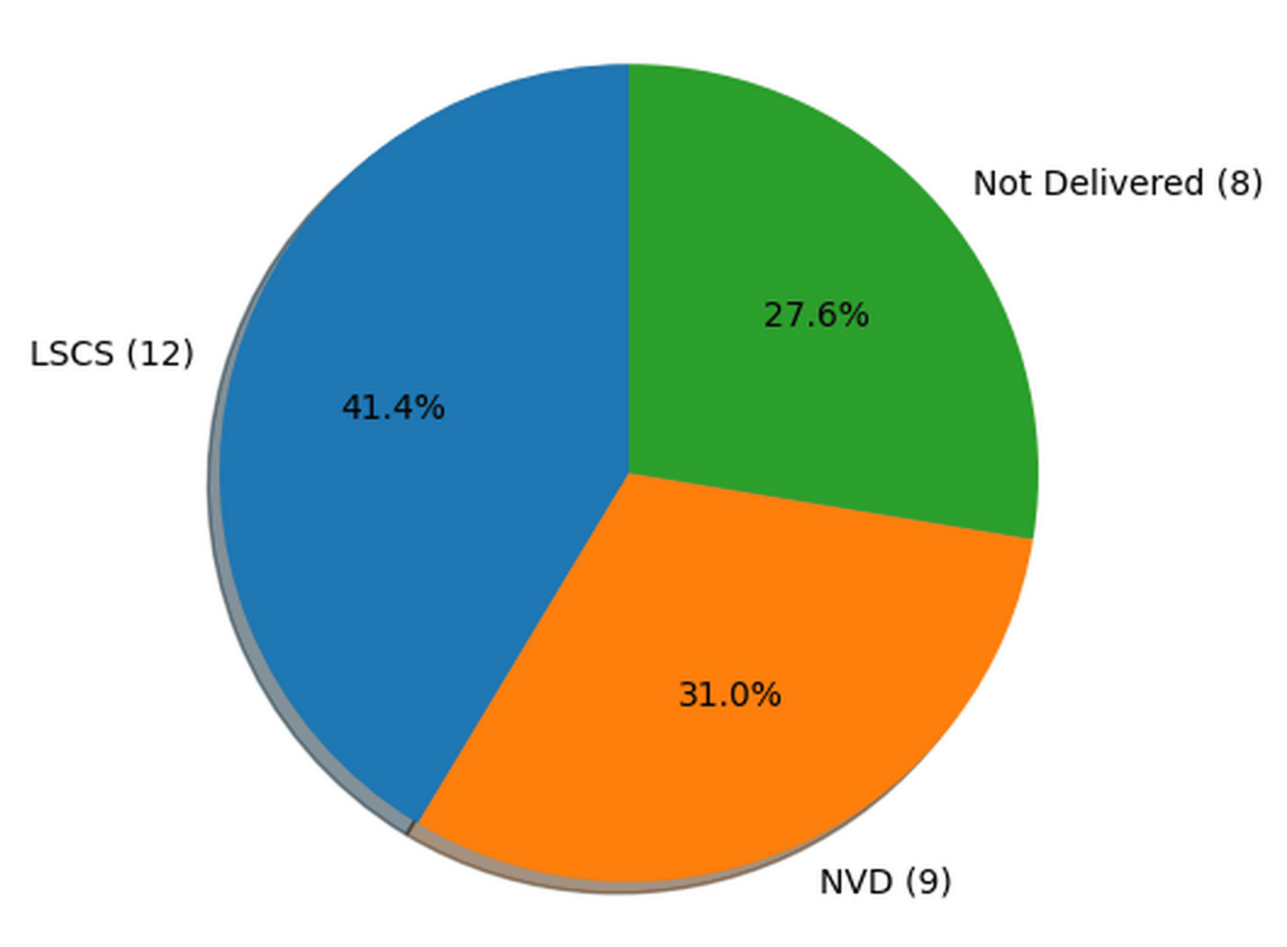
Distribution of cases by mode of delivery (n=29) LSCS: Lower Segment Cesarean Section

With respect to the mode of delivery, 12 cases (41.4%) underwent lower segment cesarean section (LSCS), nine cases (31.0%) had normal vaginal delivery (NVD), and eight cases (27.6%) remained undelivered at the time of death (Figure 5).

Referral status and place of death

Most women were referred from peripheral healthcare facilities to higher centers (n = 24, 82.8%). Of the 29 cases, 17 women (58.6%) died during treatment at the hospital, while 12 (41.4%) were brought dead to the hospital.

Cause of death

**Figure 6:**
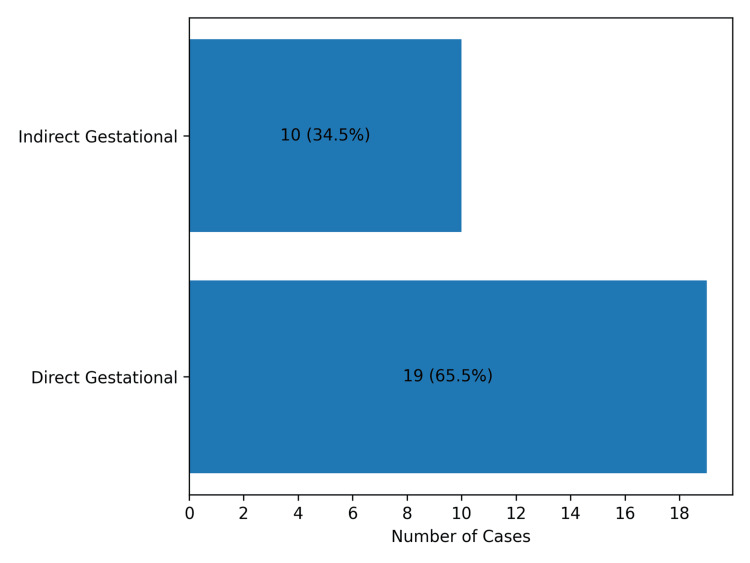
Distribution of cases by direct vs. indirect gestational causes of maternal death (n=29)

**Figure 7:**
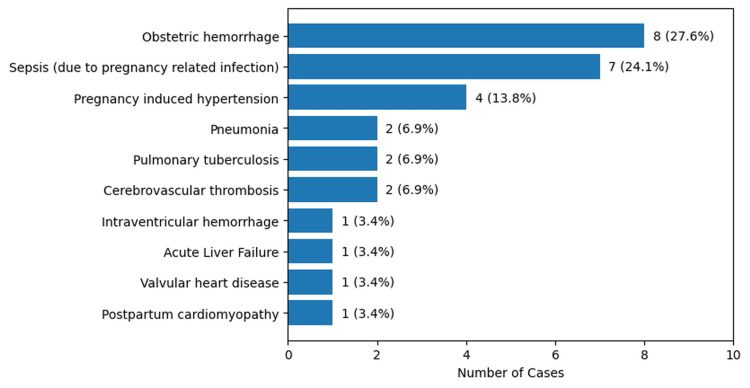
Distribution of cases by causes of maternal death (n=29)

Out of the 29 cases, 19 (65.5%) died due to direct gestational causes, while 10 (34.5%) died due to indirect causes (Figure 6). Obstetric hemorrhage was the leading cause, accounting for eight cases (27.6%). This category included postpartum hemorrhage (n = 3, 10.3%), antepartum hemorrhage (n = 2, 6.9%), hemorrhage due to rupture of the uterus (n = 2, 6.9%), and hemorrhage due to rupture of ectopic pregnancy (n = 1, 3.4%). Sepsis (due to pregnancy-related infection) was the second most common cause, responsible for seven cases (24.1%), followed by pregnancy-induced hypertension in four cases (13.8%). Other causes included pneumonia, pulmonary tuberculosis, and cerebrovascular thrombosis, each contributing two cases (6.9%). Intraventricular hemorrhage, acute liver failure, valvular heart disease, and postpartum cardiomyopathy were observed in one case each (3.4%) (Figure 7).

## Discussion

The present autopsy-based study provides important insights into the epidemiological characteristics, obstetric profile, and causes of maternal deaths in a tertiary care setting. Analysis of these cases helps in identifying patterns and determinants of maternal mortality, which are essential for improving maternal health services and preventing avoidable maternal deaths.

In the present study, the median age of the females was 29 years, with the majority of maternal deaths occurring among women aged 20-30 years (n = 20, 69.0%), which corresponds to the peak reproductive age group. A systematic review conducted by Tajvar M et al. reported that maternal age below 18 years or above 34 years is a significant determinant of maternal mortality [7]. Similarly, a systematic review by Yakubu et al. observed that both young maternal age (<20 years) and advanced maternal age (≥30 years) are positively associated with an increased risk of maternal mortality [4]. Furthermore, Marchie et al. identified early marriage and early pregnancy as important predictors of maternal mortality, which are often influenced by socio-cultural factors and religious practices [8]. Younger mothers are particularly vulnerable because they may be physically immature, with inadequately developed pelvic structures, which predisposes them to obstructed labor and related complications that may result in severe morbidity or death [9]. Although both young maternal age and advanced maternal age are associated with an increased risk of maternal mortality, pregnancies among women of advanced maternal age carry an even greater risk. This is because increasing maternal age significantly raises the likelihood of both direct and indirect obstetric complications such as hemorrhage, placenta previa, diabetes, and hypertension [4].

The majority of cases in the present study belonged to the lower middle class (n = 14, 48.3%), followed by the middle class (n = 12, 41.4%). This distribution highlights the significant representation of socioeconomically weaker groups among the study population. A systematic review by Tajvar et al. identified low family income as an associated factor in nearly 87% of the included studies, underscoring the strong relationship between socioeconomic status and maternal outcomes [7]. Income has been shown to correlate negatively with maternal mortality, with lower income groups experiencing a disproportionately higher risk. Financial constraints can limit access to adequate antenatal care and institutional delivery services, both of which are critical for reducing the risk of maternal death [10].

A vast majority of women in our study were from rural areas (n = 24, 82.8%). Similar observations have been reported in previous studies. Ward ZJ et al. found that maternal mortality was significantly higher among women residing in rural areas and among those with lower levels of education [11]. Similarly, Harrington KA et al., in a study conducted in the United States, also reported higher maternal mortality among women living in rural regions [12]. The increased risk of adverse maternal outcomes in rural areas is likely multifactorial.

In developing countries such as India, women in rural areas often have lower levels of education, and several studies have demonstrated a significant association between maternal educational status and maternal mortality [4,8]. Additionally, many rural regions lack adequately equipped health facilities and skilled birth attendants, which may delay the management of obstetric complications [12]. Transportation barriers and long distances to healthcare facilities are also common in rural settings, resulting in delays in reaching appropriate medical care during obstetric emergencies [12]. In the present study, most women were referred from peripheral healthcare facilities to a higher center (n = 24, 82.8%), and a considerable proportion of women were brought dead to the hospital (n = 12, 41.4%), highlighting the impact of delays in accessing timely obstetric care and the lack of adequate health facilities in rural areas. Furthermore, early marriage and early pregnancy are more prevalent in rural areas, which further increases the risk of maternal mortality [8,13]. These findings highlight the need to strengthen healthcare infrastructure in rural areas, promote women’s education, and ensure timely access to quality obstetric care.

In the present study, the majority of cases had attained education up to middle school (n = 9, 31.0%), followed by primary school (n = 8, 27.6%), while five cases were illiterate. A systematic review by Yakubu et al. reported a negative correlation between maternal education and MMR [4]. The role of education in reducing MMR is multidimensional, as it enhances awareness and enables women to make informed reproductive choices, seek timely antenatal and postnatal care, and opt for institutional deliveries. Furthermore, education promotes financial independence and autonomy, allowing women greater control over their health and fertility. Given that literacy rates remain relatively low in low- and middle-income countries such as India, improving female education may significantly contribute to the reduction of maternal mortality [4,7].

A substantial proportion of maternal deaths in our study occurred during the postpartum period (n = 22, 75.9%). Similar observations have been reported in previous studies. A review by Li XF et al. reported that more than 60% of maternal deaths occur during the postpartum period, with approximately 45% occurring within the first 24 hours after delivery and over 65% within the first week [14]. Similarly, Dol J et al., in a systematic review, observed that the highest proportion of maternal deaths occurred on the first day postpartum (48.9%), followed by deaths occurring between days 2 and 7 (24.5%) and between days 8 and 42 (24.9%) [15]. These findings highlight the postpartum period, particularly the early postpartum days, as a critical window for maternal survival. Therefore, ensuring timely access to quality postpartum care, including regular monitoring and prompt management of complications during the first 42 days after delivery, is essential for improving maternal outcomes and reducing maternal mortality [14,15].

The findings of the present study indicated that 12 cases (41.4%) were associated with LSCS, while nine cases (31%) followed NVD. Similar observations have been reported in previous studies. A meta-analysis conducted by Mascarello KC et al. demonstrated that women undergoing cesarean section had approximately three times higher risk of maternal mortality compared with those who had vaginal delivery [16]. Similarly, Sobhy et al., in a systematic review conducted in low- and middle-income countries, reported that the risk of maternal death among women undergoing cesarean section was 7.6 per 1000 procedures [17]. The higher risk associated with cesarean delivery may be attributed to surgical complications such as hemorrhage, infection, thromboembolism, and anesthesia-related complications, as well as the fact that cesarean sections are often performed on women with high-risk pregnancies or obstetric emergencies. Therefore, cesarean sections should be performed with appropriate indications and adequate surgical safety measures, ensuring that the benefits of the procedure outweigh the potential risks.

In this study, 65.5% (n = 19) of maternal deaths were attributed to direct obstetric causes, while 34.5% (n = 10) resulted from indirect causes. Among the direct causes, obstetric hemorrhage was the leading contributor, accounting for eight cases (27.6%), followed by sepsis (n = 7, 24.1%), and pregnancy-induced hypertension (n = 4, 13.8%). These findings are comparable with those reported in previous studies. Say L et al. reported that approximately 73% of maternal deaths worldwide between 2003 and 2009 were due to direct obstetric causes, while indirect causes accounted for 27.5% of deaths. In their analysis, obstetric hemorrhage was responsible for 27.1% of maternal deaths, followed by hypertensive disorders and sepsis, which accounted for 14% and 10.7% of deaths, respectively [18]. Similarly, Creswell et al. observed that globally the most common cause of maternal death was hemorrhage (27%), followed by indirect obstetric causes (23%) and hypertensive disorders (16%). They also observed that the proportion of deaths due to hemorrhage varied considerably by region and was highest in sub-Saharan Africa, Western Asia, and Northern Africa [19]. Montgomery AL et al. also reported that the majority of maternal deaths were due to direct obstetric causes (81.8%), with nearly one quarter of maternal deaths attributed to obstetric hemorrhage, while 15% were due to indirect causes [20].

The present study has several strengths as well as limitations. Being an autopsy-based analysis, it allows a more accurate determination of the underlying causes of maternal deaths and helps minimize misclassification compared with studies based solely on clinical records. The study also spans a period of more than seven years, providing useful insights into maternal mortality patterns in the region. However, the findings should be interpreted in light of certain limitations. The study was retrospective in nature and conducted at a single tertiary care center with a relatively small sample size, which may limit the generalizability of the results. Additionally, as this was an autopsy-based study including only maternal deaths subjected to medico-legal autopsy, the study population represents a selective subgroup and may not reflect the full spectrum of all maternal deaths, particularly institutional cases not undergoing autopsy, thereby introducing selection bias. Furthermore, detailed information regarding various other determinants, such as antenatal visits, gravida/parity, access to clean water, sanitation, electricity, hemoglobin status, and referral delays, was not consistently available, restricting a comprehensive evaluation of the broader determinants influencing maternal mortality. Owing to the limited sample size, advanced inferential statistical analyses, subgroup comparisons, and trend analysis were not performed, which may restrict deeper exploration of associations between maternal characteristics and causes of death.

## Conclusions

Maternal mortality continues to be a major public health concern, particularly in low- and lower-middle-income countries. Despite sustained global efforts, progress remains inadequate to achieve the SDG target of reducing the global MMR to below 70 per 100,000 live births by 2030. The findings of this autopsy-based study offer valuable insights into the determinants and underlying causes of maternal mortality in a tertiary care setting.

The study underscores that maternal mortality is influenced not only by biomedical factors but also by broader social determinants, including access to healthcare services, educational status, and socioeconomic conditions. Addressing maternal mortality, therefore, requires a comprehensive and multidisciplinary approach. Strengthening healthcare infrastructure, improving referral linkages and emergency transport systems, and ensuring timely access to quality antenatal, intrapartum, and postpartum care are essential measures. In addition, promoting female education and addressing socioeconomic disparities can further enhance the utilization of maternal healthcare services. Addressing these determinants through coordinated public health strategies is essential to accelerate progress toward achieving the SDGs and improving maternal survival.
